# Apoptosis at Inflection Point in Liquid Culture of Budding Yeasts

**DOI:** 10.1371/journal.pone.0019224

**Published:** 2011-04-27

**Authors:** Toshiyuki Hagiwara, Takashi Ushimaru, Kei-ichi Tainaka, Hironori Kurachi, Jin Yoshimura

**Affiliations:** 1 Graduate School of Science and Technology, Shizuoka University, Hamamatsu, Japan; 2 Daiichi Sankyo Co., Ltd., Fukuroi Research Center, Fukuroi, Japan; 3 Graduate School of Science and Technology, Shizuoka University, Oya, Shizuoka, Japan; 4 Department of Systems Engineering, Shizuoka University, Hamamatsu, Japan; 5 Marine Biosystems Research Center, Chiba University, Uchiura, Kamogawa, Chiba, Japan; 6 Department of Environmental and Forest Biology, State University of New York College of Environmental Science and Forestry, Syracuse, New York, United States of America; University of Maribor, Slovenia

## Abstract

Budding yeasts are highly suitable for aging studies, because the number of bud scars (stage) proportionally correlates with age. Its maximum stages are known to reach at 20–30 stages on an isolated agar medium. However, their stage dynamics in a liquid culture is virtually unknown. We investigate the population dynamics by counting scars in each cell. Here one cell division produces one new cell and one bud scar. This simple rule leads to a conservation law: “The total number of bud scars is equal to the total number of cells.” We find a large discrepancy: extremely fewer cells with over 5 scars than expected. Almost all cells with 6 or more scars disappear within a short period of time in the late log phase (corresponds to the inflection point). This discrepancy is confirmed directly by the microscopic observations of broken cells. This finding implies apoptosis in older cells (6 scars or more).

## Introduction

Aging of cells is an extremely complex phenomenon, which is closely related to their proliferation (reproduction, division) and differentiations. Budding yeast is commonly use as a model organism for cell aging and senescence [1]–[4], because of the asymmetric cell division and budding scars. Here a mother cell buds off (make birth) a daughter cell, leaving one bud scar. The number of scars indicates how many daughter cells are produced from that mother [5], [6]. Therefore, the stage of budding yeast is defined by the number of scars, or division (fission, budding). We here study the stage-structure of a budding yeast population in a liquid culture.

The life span based on stage (No. scars) is called replicative life span (RLS) [7]. The age defined by the absolute time after birth (budding) is called the chronological life span (CLS) [8]. Since the stage (the number of scars) proportionally correlates with the age (an absolute time), the stage is another functional measure of “aging” in budding yeasts. In the budding yeast, the RLS is known to be about 20–30 when cultured individually-isolated on an agar medium [9]–[11]. However, the individually-isolated culture condition does not represent the real RLS in natural growth conditions in a liquid culture.

In the natural conditions, budding yeast grows in a population of many cells with various stages. It can be represented by either a liquid culture or a culture on agar medium (surface culture). A wild yeast population in a culture should consist of cells with almost all possible stages or time. Since Hartwell's seminal works [12]–[14] the stage structure of a cultured population has been studied extensively from both mathematical and empirical approaches [12]–[19]. Various stage-specific changes in growth and reproduction are found along growth phases. For example, the first budding of daughter cells (stage 0) in a late log-phase is reported to delay compared with that in an early log-phase, resulting that the proportion of daughter cells increases in a late log-phase [19]. Unfortunately, the measurement of the stage of a single cell is practically impossible in liquid cultures. Therefore, both the RLS and CLS of individual yeast in the cultures are virtually unknown.

**Figure 1:**
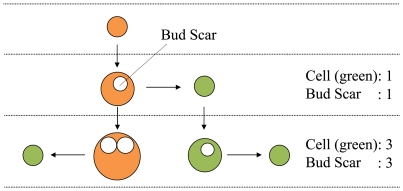
A conservation law of cells and bud scars in budding yeasts. After a few hours of culturing, the total number of cells becomes about equal to the total number of bud scars in a liquid culture, because a cell (orange) leaves one bud scar (white circle) when reproduces a daughter cell (green).

In the budding yeast, we have a simple rule: one new cell (cell division) produces one bud scar on a mother cell. From this simple rule, we get a unique conservation law for a liquid culture of budding yeast (Fig. 1): the population size (the total number of cells) is nearly equal to the total sum of scars of all cells [18]–[19]. After a few hours of seeding, the number of the original cells becomes less than 0.05% of the total number of cells. Therefore, the original cells become always negligible in this conservation law. We should note here that this conservation law has a very important assumption: all the cells are immortal. If many old cells die in a culture, the total number of cells becomes more than that of scars. Oppositely, if many daughter cells (stage 0) die, the total number of cells becomes less than that of scars.

Based on this conservation law, we can predict the exact stage structure (i.e., the distributions of scars) of a yeast population. By counting the number of scars in cells in several different phases, we investigate the stage dynamics of budding yeast in liquid cultures. Traditionally it is difficult to count the number of bud scars in a cell with high accuracy. We successfully count the number of scars a yeast cell by recording its z-axis slices under microscope. In the result section, we report a large discrepancy found between the observed and expected stage structures at the end of the log-phase. The number of cells older than 5 is extremely fewer than the expected from the conservation law. From the mathematical model, we predict that the deaths of old cells are concentrated in a very short period at the end of the log-phase. We then scrutinize the liquid culture and found many broken/tiered cells with many scars. This implies the programmed death or apoptosis depending on the cumulative number of cell division in this phase. We discuss some biological significances of this unexpected apoptosis in a liquid yeast culture.

## Results and Discussion

**Figure 2:**
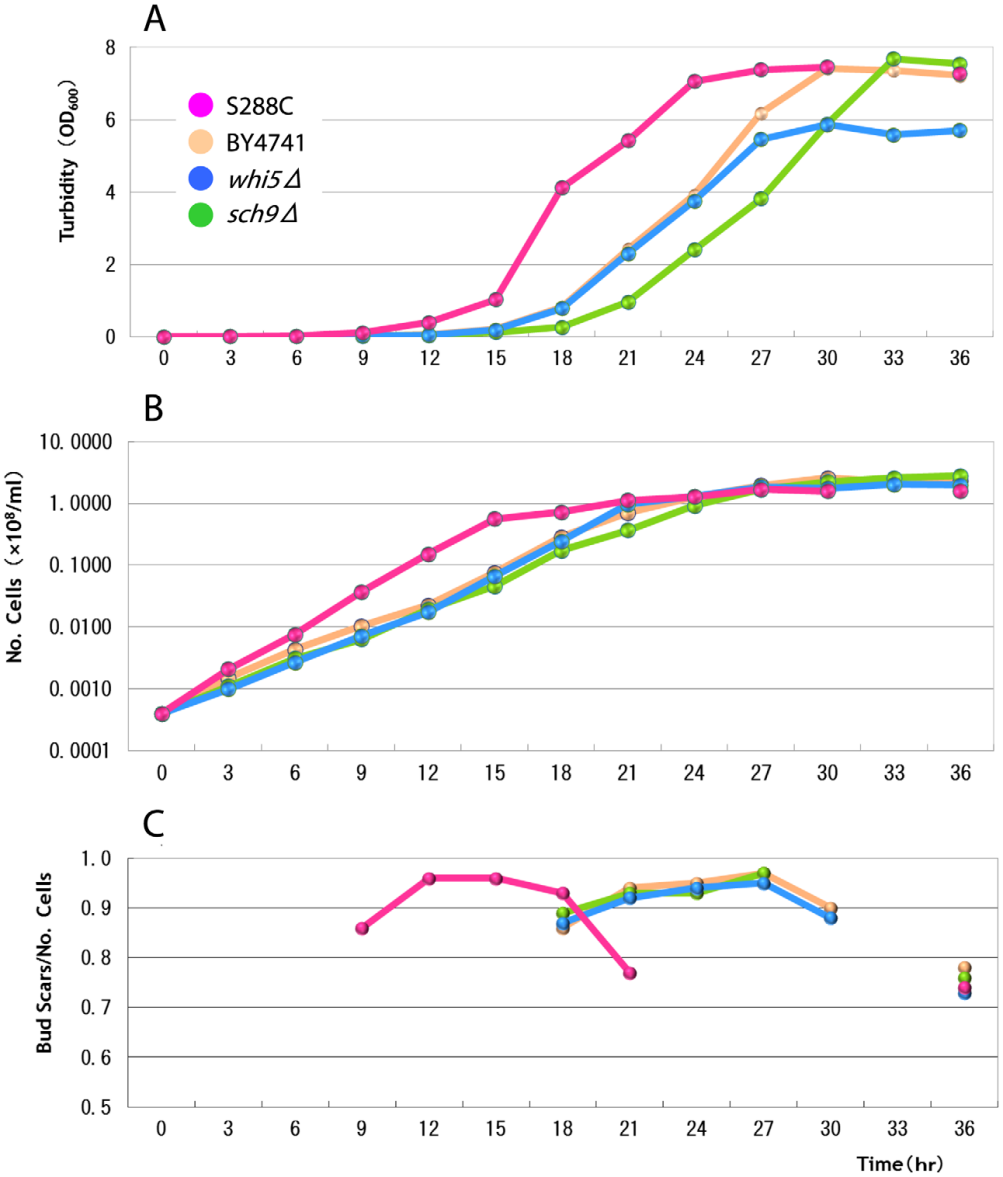
Temporal changes in turbidity, number of cells and average number of bud scars per cell. A: The turbidity indicative of proliferation. B: The number of cells indicating population growth. C: The average number of bud scars per cell indicating the breakdown in the conservation law. (Data are shown in Tables S1, S2, S3 and S4.). This value should be unity (1.00) if the conservation law is satisfied. The delayed population growth is seen in all mutant strains. Similarly the breakdown in the conservation law is also delayed in all mutant strains, but the final levels of breakdown (at 36 hours) are similar in all the strains.

The population growth profiles of all strains are usual sigmoid curves with about 10^8^ cells at saturation (Fig. 2). The wild strain S288C saturates at about 21 hours, while all other nutrient deficient strains at about 30 hours showing some constant delay in growth profiles (Fig. 2A and 2B).

Fig. 2C shows the discrepancies from the conservation law. Here the average number of scars/cell (No. scars) is 1 if there is no discrepancy, less than 1 if older cells die frequently, and more than 1 if younger cells die more. The discrepancies from the conservation law are minimized (No. scars  =  ca. 0.95) during the log phases (12–18 hours in S288C strain; 21–27 hours in the other strains) (Fig. 2C). The discrepancies are radically increased at the end of the log phase (No. Scars  =  ca. 0.77 at 21 hours in S288C and 0.88∼0.92 at 30 hours in the rests). At 36 hours, the ratio of conservation law decreases to about 0.73∼0.78.

**Figure 3:**
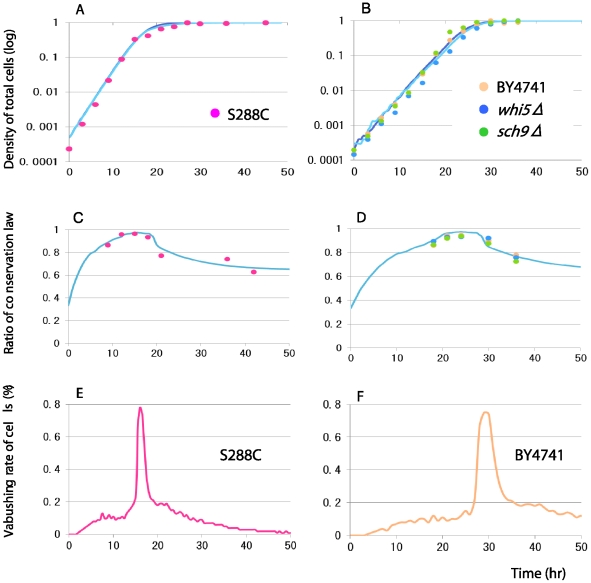
Comparisons of simulation results (curves) of lattice gas model with experimental data (plots). Four strains of budding yeasts are used (pink: S288C; veige: BY4741; blue: *whi5Δ*; green: *sch9Δ*). A,B: The population growths. C,D: The ratio of conservation law (the number of scars per cell) asumming various critical stage limits, above which all cells die. The stage limit  = 6 is depicted. The experimental data fits with the simulations when the stage limit is 6 scars. E,F: The vanishing rate of cells/all cells/hour. The maximum frequency of apoptosis is less than 0.8% at the peak, but quickly converges to zero afterwords, indicating that the observations of apoptosis is nearly impossible except in this short period and fairly difficult even within this period. All simulation data are based on the ensemble averages of 100 trials. The parameters are *r_m_* = 0.7, *r_md_* = *r_m_* (0∼*x* hours) or 0.0 (>*x* hours), where *x* = 15 for S288C and 25 for all mutant strains. The initial proportion of daughter and mother is 8: 2. The mortality rate *m_n_* is 0.05 (0∼*y* hours) or 1.0 (*y* hours), where *y* = 18 for S288C and 27 for all mutant strains. The density dependent death (apoptosis) caused by the stage limit is introduced at *y* = 15 hours in strain S288C (C)and 25 hours in all mutant strains (D).

In order to evaluate the discrepancy from the conservation law, the temporal dynamics of the average number of scars are estimated using the mathematical population model. For both the wild S288C and the deficient strains, the growth parameters are estimated from the logistic growth profiles (Figs. 2B, 3A and 3B). The abrupt change in the number of scars at 21 hours in the wild strain S288C (Fig. 2C) cannot be reproduced by the mathematical model with any gradual change in parameter conditions. A sudden introduction of mortality seems necessary to reproduce abrupt changes in the average number of scars. The predicted scar profiles become similar to those of the wild strain S288C when the stage (scar)-dependent mortality thresholds, *ds* = 1, for stages 5∼10 are introduced at 15 hours together with *ds* = 0.05 during 0∼15 hours (Fig. 3C). When the mortality threshold *ds* = 6, the dynamics of average scars are matches with the experimental data quite well for the wild S288C strain (Fig. S1). For the deficient strains, the predicted scar profile matches well with the observed scar dynamics when the mortality threshold is introduced at 30 hours (Fig. 3D).

From the predicted scar profiles, we estimate the temporal change of vanishing rates of old cells. Most old cells disappear right after the abrupt mortality is introduced (at 15 hours for the strain S288C in Fig. 3E, at 30 hours for the nutrient deficient strains in Fig. 3F). This means that the observations of dead broken cells are almost impossible except those peak times. Even at the peak times, the observations of destroyed cells are fairly difficult since the rates of vanishing cells at the peak are less than 0.8% of all cells.

**Figure 4:**
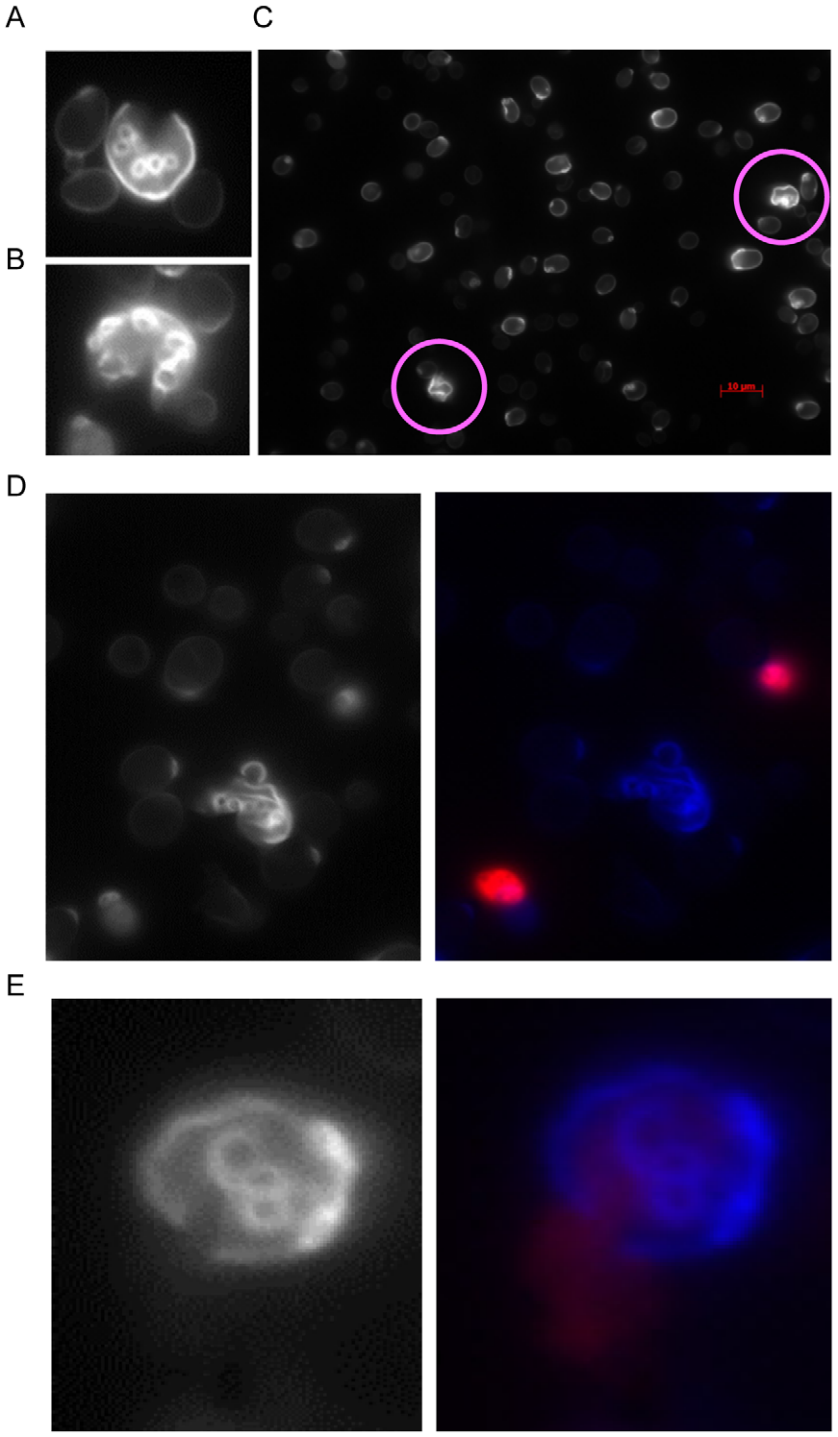
Photo images of broken cells. A, B: Broken cells with many scars (at 21 hours in S288C). A part of cell walls are lost. C: A preparation images of many cells. Two broken cells (red circles) are found between many healthy cells (thin whitish images). D: Images of a broken cell and two unbroken dead cells (right: normal view; left: dyed view). The ordinary dead cells (apoptosis) are dyed by propidium iodide (DNA material: red) and the broken cell (blue: dyed by dapi), from which DNA material is lost. E: An image of a broken cell (blue), where DNA material (red) is leaking from the breakage of the cell wall (right: normal view; left: dyed view).

We then observe the vanishing cells by microscopes sampling at about the peak time (Fig. 3E and 3F). Many physically broken cells are found at those phases (Fig. 4A). In the broken cells, the part of surface membrane is broken physically (blue dye in Fig. 4B) that is different from the usual apoptosis where the entire membrane of a cell is weakened (red dye in Fig. 4B). Fig. 4C shows that the DNA material is leaking from the broken membrane of a cell. We should also note that the coincidence of these observations of broken cells (Fig. 4) and the mathematical predictions of the peak times (Fig. 3E and 3F) indicates that the broken cells are not caused by physical damages during the slide preparation and other treatments. We could scarcely find broken cells in the other stages.

The current result is similar to the paradoxical density effect in the log phase, where the proportion of daughter cells increases suddenly [19]. In their case, the sudden suspension of reproduction in daughter cells causes the increase in daughter proportion in a population. In the current case, the sudden deaths in older cells result in the abrupt change in the average numbers of scars. Thus some qualitative changes of a population are associated in both cases of the abrupt changes in a population.

In an isolated culture, the longevity of budding yeast is considered 20–30 scars. This study is the first report of the stage dependent cell death only in a population. However, the sudden deaths of 6 scars in a liquid population are considerably shorter. In natural conditions, the longevity may have to be considered about 6 (scars). This unexpected apoptosis may be controlled by a process of senescence.

Our findings of the abrupt deaths of old cells (>5 staged cells) are quite different from those of traditional apoptosis, where younger cells are dissolved by the fusion of cell membrane. Rather these cells are tiered up that is similar to the symptom of necrosis. This sudden death of old cells can be considered as a new type of apoptosis only occurring selectively in the cells with 6 or more scars in a population.

## Materials and Methods

### Experimental methods

The budding yeasts used are two wild strains and their two knockout mutants: the wild strain S288C (*MATa mal gal2*) [20], its wild auxotrophic (nutrient requiring) strain BY4741 (*MATa ura3Δ0 leu2Δ0 his3Δ1 met15Δ0*) [21], their knockout mutants*whi5Δ* (G1/S progression inhibitor Whi5 deficient) [22], *sch9Δ* (nutrient responsive kinase Sch9 deficient) [23]. The stage dynamics are estimated by a multi-stage yeast population model. This model is expanded from the three stage version of lattice gas model [19]. The discrepancy from the conservation law is estimated by introducing stage-specific mortalities in the model.

We sample about 1–3 ml of the yeast every three hours for 36 hours from the beginning. As a trial, we also sample at 48 hours at one test for S288C. We evaluate the proliferation performance by measuring the number of cells and turbidity (OD_600_). The number of cells and turbidity are measured every three hours.

We prepare the preparation slides at 9, 12, 15, 18, 21 and 36 hours for the S288C strain. For the other strains (BY4147, *whi5Δ*, *sch9Δ*), the preparation slides are made at 18, 21, 24, 27, 30 and 36 hours. These slides are used to measure the stage distribution of yeast cells (the frequency distribution of the number of scars), from which the distribution of mother and daughter cells are estimated. The numbers of scars are counted for 500 hundred cells except 21 hours in the mutant strain *whi5Δ*. The detail experimental procedure is shown in Text S1. The counting data are shown in Tables S1, S2, S3 and S4 for all strains.

### Theoretical analyses

#### Simulation model

For mathematical analyses, we apply a lattice gas model that is suitable for studying the population growth with density effects [19]. In this model, the total number of lattice sites is kept constant, i.e. carrying capacity *K*. The carrying capacity *K* represents the total volume of the medium in each culture. The simplest lattice gas model is the one-stage model with stage X. Each lattice sites are either occupied (X site) or empty (O site). The density effects are automatically built in by the number of empty sites. The empty sites are the only sites on which a cell can propagate. The total number of cells never increases beyond the total number of lattice sites.

Here we develop a multi-stage lattice gas model modifying the above one-stage model. The cell cycle of budding yeast is classified into two classes: daughter (D) and mother (M). The daughters (D) have no scar, while mothers (M) have at least one scar. The daughter (D) is further divided into the immature (D_i_) and the matured (D_m_) based on the size of cells. The cell size of the immature daughter (D_i_) is smaller than that of mothers, while the cell size of the matured is about the same size of mothers. The mothers are also classified into different stages based on the number of scars. In mothers, stage *n* means the cell has *n* scars.

The current model is considered the extended version of the multi-stage lattice gas model with mother and two-daughter stages (Tainaka Model) [19]. A distinct point of the current model is the inclusion of the stages (No. scars). The current model is also considered the extension of the stage-structured dynamic model based on the number of scars (Hamada model) [17]. Thus the current model is considered the unification of both Tainaka model and Hamada model.

We study the population dynamics of stage distributions while the density effects start acting until the population growth is ceased (see Text S2 for simulation procedure). We specifically focus on the log phase of the logistic growth. The cells (lattice sites) are classified into the following multi-stages: D_i_: the immature daughter (no scars and small cell); D_m_: the matured daughter (no scars and large cell); M_n_: mother cell with *n* scars (*n*≥1); O: empty site (no cell).

The reaction equations of all cells are as follows:
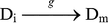
(1a)

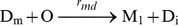
(1b)

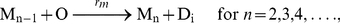
(1c)

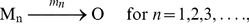
(2)


Reactions (1a)-(1c) and (2), respectively, represent growth of daughter, reproduction of daughter, reproduction of stage-*i* mother, and death of mother, where *g*, *r_md_*, *r_m_* and *m_i_* are reaction rates for respective processes. Note that the reproduction rate of matured daughter, *r_md_*, becomes zero ( = 0) only when the daughter-specific density dependent effects are introduced at the late log-phase (e.g., 15 hours in S288C culture); otherwise it kept the same (*r_md_* = *r_m_* = 0.3) [19].

We also perform the test without the daughter-specific density effects, such that *r_md_* = *r_m_* for all times. The results without the density effects are almost identical with only a slight quantitative difference from those with the daughter-specific density dependence. Furthermore, the current empirical results (unpublished data) also reconfirm the daughter-specific density effects [19]. Therefore, we here report the results with the daughter-specific density effects.

The reproduction rate of mother, *r_m_*, is assumed to be independent of the stage of mothers (i.e., the number of scars) [14]. Each lattice site is either empty (O) or occupied by one of stages in the three classes (D_i_, D_m_, and M_n_ for *n* = 1,2,…). The total number of lattice sites is assumed to be equal to the carrying capacity *K*.

We here introduce the stage-specific mortalities in the following manner. First, the density independent mortality is kept at *m_n_* = 0.05 for all stages. The stage-specific density dependent mortality (*m_n_* = 1.0) for a given stage limit (*n*>*n_limit_*) is introduced at the late log phase (e.g., 15 hours in S288C culture). We vary this apoptosis limit and compare the results with the experimental data, such that *n_limit_* = 5,6,…,10.

#### Differential equations

Mathematically the above population can be calculated by the following set of rate equations.
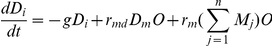


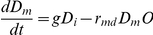


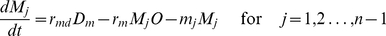


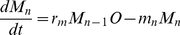
(3)


The set of differential equations comes from reactions (1a)–(1c) and (2). For example, the last equation in the Eq. (3) can be derived from reactions (1c) and (2). The total population size (density) D of daughter is the sum of D_i_ and D_m_, while the density M of mothers is the sum of all M_n_ (for n = 1,2,…,n,…).

We can confirm that the same results are obtained by both simulation and mathematical analyses. Note that assuming the conservation law (no stage-dependent apoptosis) our model is consistent with the previous three-stage models.

For matching the experimental data with theoretical analyses, we use the simulation methods with varying parameter and initial conditions (see Text S2 for simulation procedure). For the evaluation of qualitative results, we compare the mathematical analyses with simulation results to confirm that the simulation is indeed correctly performing the supposed procedures.

## Supporting Information

Figure S1 **Comparisons of simulation results (curves) with experimental data (plots).** Four strains of budding yeasts are used (pink: S288C; veige: BY4741; blue: *whi5Δ*; green: *sch9Δ*). A,B: The ratio of conservation law (the number of scars per cell) asumming various critical stage limits, above which all cells die. The stage limit is indicated as bud scar limit in the figures. C,D: The same as A, B, but when the stage-limit is 6 only is depicted. The experimental data fits with the simulations when the stage limit is 6 scars. All simulation data are based on the ensemble averages of 100 trials. The parameters are *r_m_* = 0.7, *r_md_* = *r_m_* (0∼*x* hours) or 0.0 (>*x* hours), where *x* = 15 for S288C and 25 for all mutant strains. The initial proportion of daughter and mother is 8: 2. The mortality rate *m_n_* is 0.05 (0∼*y* hours) or 1.0 (*y* hours), where *y* = 18 for S288C and 27 for all mutant strains. The density dependent death (apoptosis) caused by the stage limit is introduced at *y* = 15 hours in strain S288C (A)and 25 hours in all mutant strains (B).
(TIF)

Text S1 Detail experimental procedure.
(DOC)

Text S2 Simulation procedure.
(DOC)

Table S1 Temporal change in stage compositions of budding yeast culture (Strain: S288C).
(TIF)

Table S2 Temporal change in stage compositions of budding yeast culture (Strain: BY4741).
(TIF)

Table S3 Temporal change in stage compositions of budding yeast culture (Strain: *sch9Δ*).
(TIF)

Table S4 Temporal change in stage compositions of budding yeast culture (Strain: *whi5Δ*).
(TIF)
