# Supplementary figures and images for: Apoptosis at Inflection Point in Liquid Culture of Budding Yeasts
Source: PLoS One. 2011 Apr 27;6(4):e19224. doi: 10.1371/journal.pone.0019224 (PMC3083425; doi:10.1371/journal.pone.0019224)

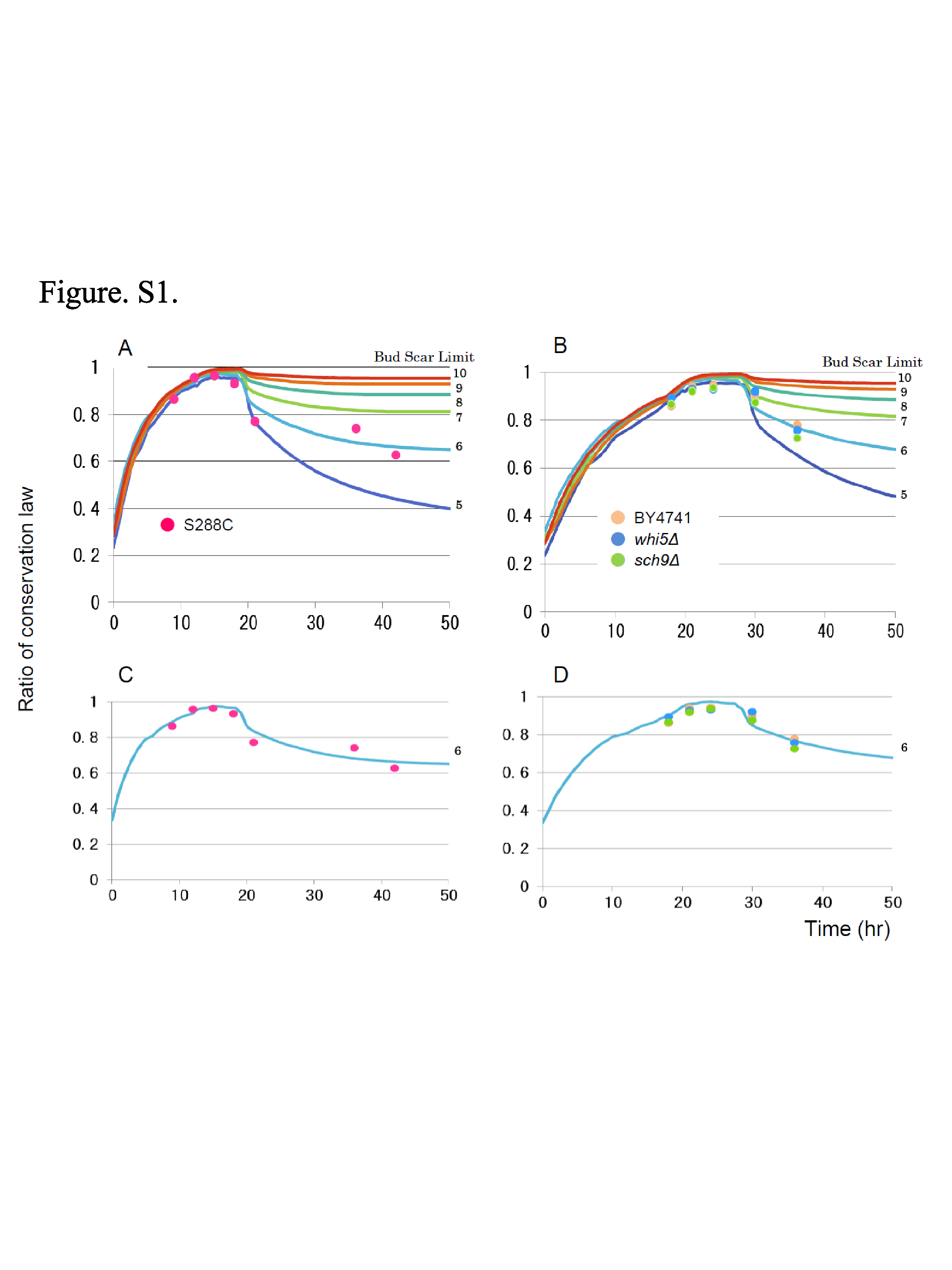

Supplement: Figure S1 — Comparisons of simulation results (curves) with experimental data (plots). Four strains of budding yeasts are used (pink: S288C; veige: BY4741; blue: whi5Δ; green: sch9Δ). A,B: The ratio of conservation law (the number of scars per cell) asumming various critical stage limits, above which all cells die. The stage limit is indicated as bud scar limit in the figures. C,D: The same as A, B, but when the stage-limit is 6 only is depicted. The experimental data fits with the simulations when the stage limit is 6 scars. All simulation data are based on the ensemble averages of 100 trials. The parameters are rm = 0.7, rmd = rm (0∼x hours) or 0.0 (>x hours), where x = 15 for S288C and 25 for all mutant strains. The initial proportion of daughter and mother is 8: 2. The mortality rate mn is 0.05 (0∼y hours) or 1.0 (y hours), where y = 18 for S288C and 27 for all mutant strains. The density dependent death (apoptosis) caused by the stage limit is introduced at y = 15 hours in strain S288C (A)and 25 hours in all mutant strains (B). (TIF) [file pone.0019224.s001.tif]

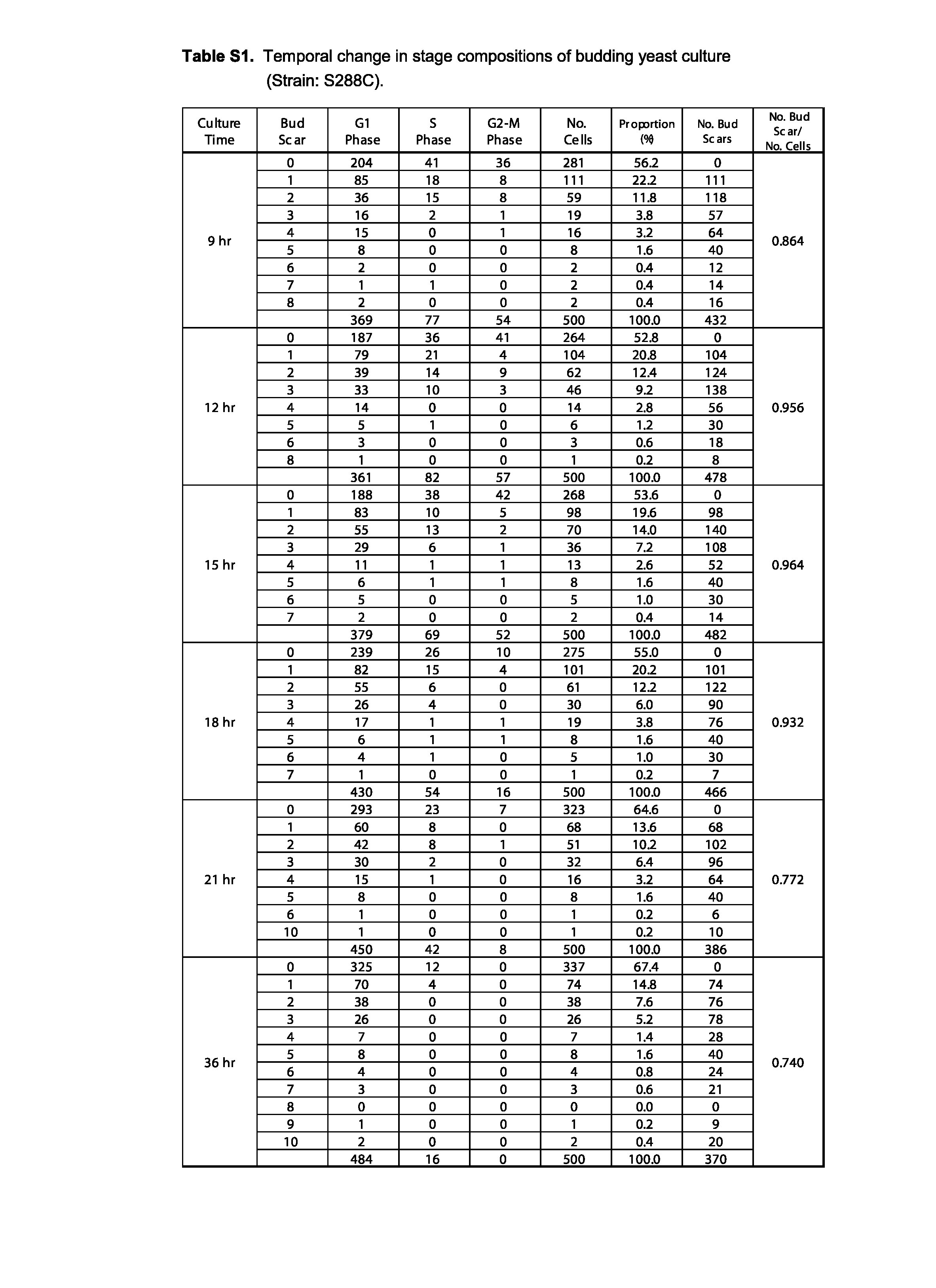

Supplement: Table S1 — Temporal change in stage compositions of budding yeast culture (Strain: S288C). (TIF) [file pone.0019224.s004.tif]

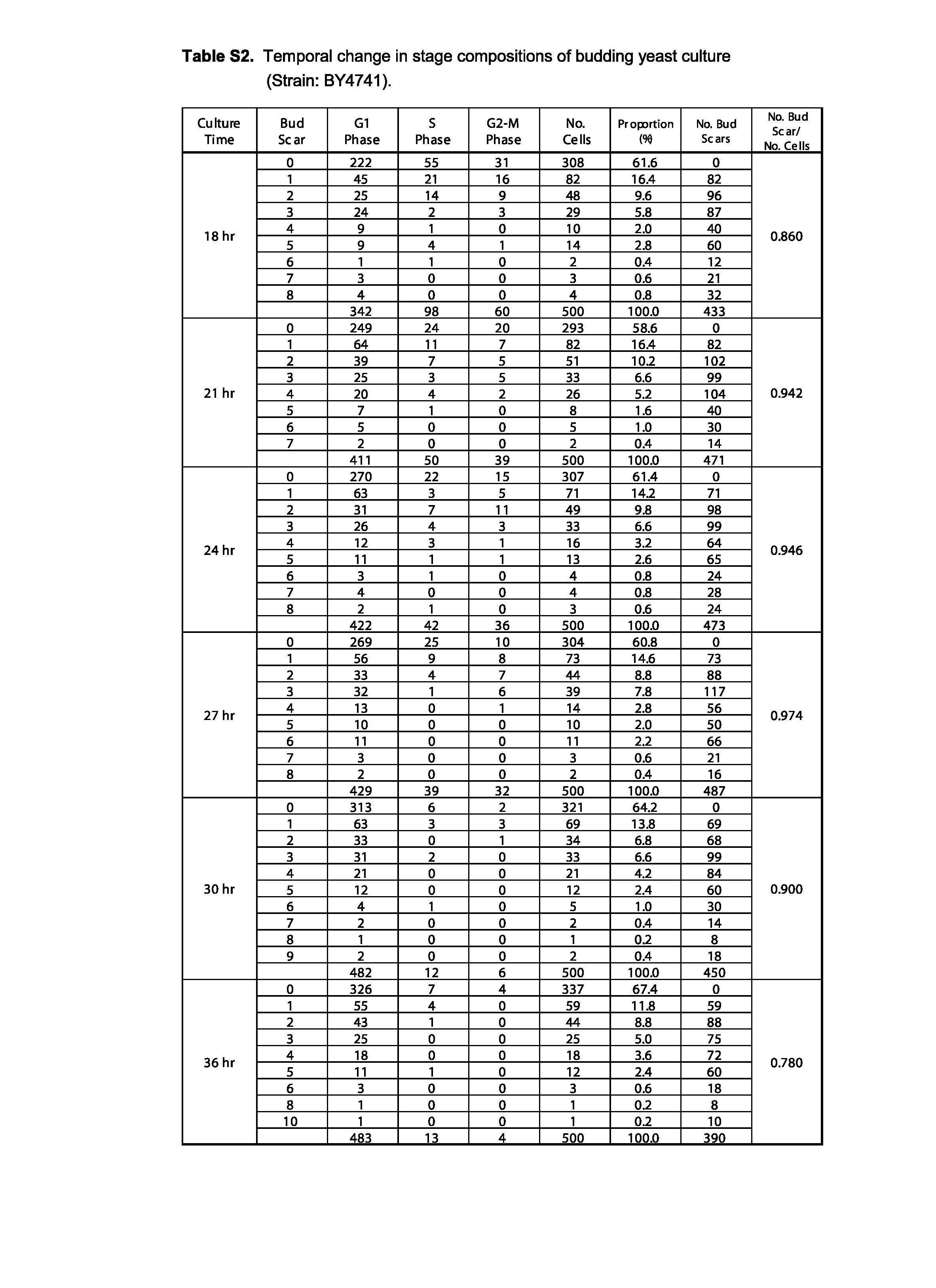

Supplement: Table S2 — Temporal change in stage compositions of budding yeast culture (Strain: BY4741). (TIF) [file pone.0019224.s005.tif]

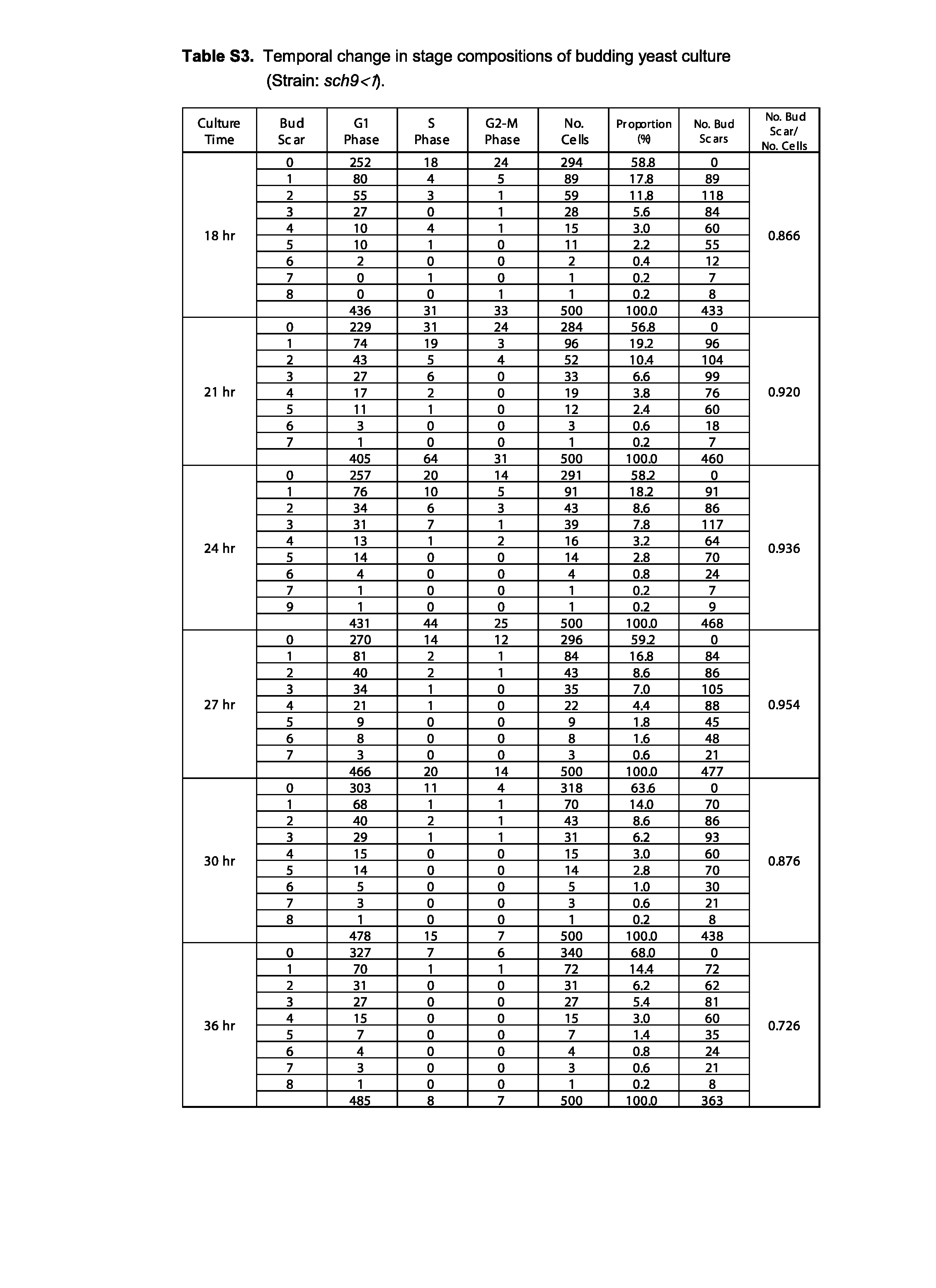

Supplement: Table S3 — Temporal change in stage compositions of budding yeast culture (Strain: sch9Δ). (TIF) [file pone.0019224.s006.tif]

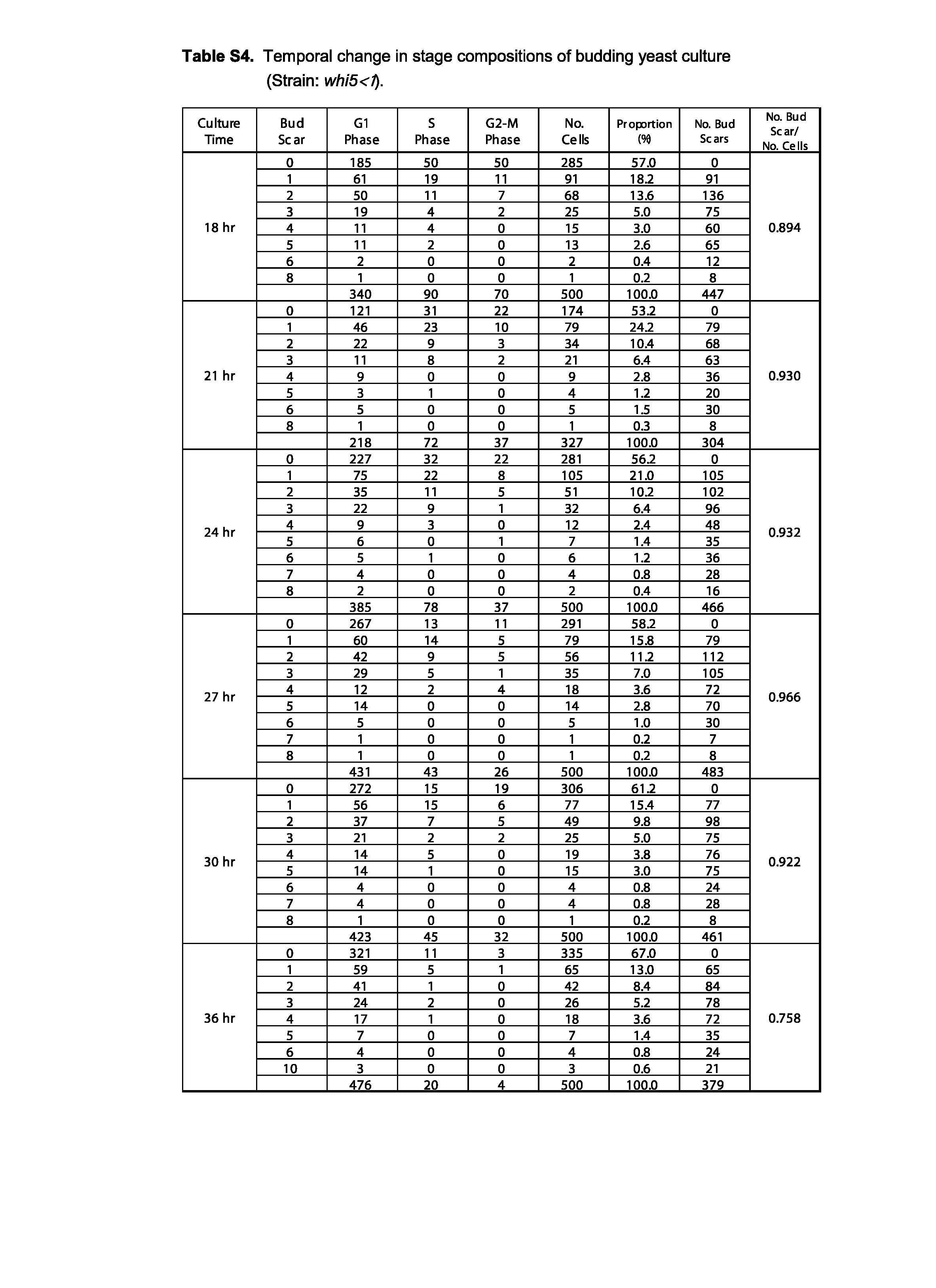

Supplement: Table S4 — Temporal change in stage compositions of budding yeast culture (Strain: whi5Δ). (TIF) [file pone.0019224.s007.tif]
