# Supplementary material for: Apoptosis at Inflection Point in Liquid Culture of Budding Yeasts
Source: PLoS One. 2011 Apr 27;6(4):e19224. doi: 10.1371/journal.pone.0019224 (PMC3083425; doi:10.1371/journal.pone.0019224)
Supplement: Text S1 — Detail experimental procedure. (DOC) [file pone.0019224.s002.doc]

Text S1.

Detail experimental procedure

The culture medium used is the SC synthetic medium added with the glucose (final concentration: 2%) and the amino acids. The yeasts are cultured in the SC synthetic medium by a shake culture 30°C. The culture condition is shaking culture with 200ml of the culture medium in a 1,000ml-Erlenmeyer flask in the incubator of 30°C. The pre-pre-cultured and pre-cultured budding yeast are adjusted to 8×10^4^/ml (final density) and the number of sowing yeast are added to the nutrient medium.

For each slide specimen, we take the Z-axis sliced photomicrographs (0.25 μm thickness). The number of bud scars on each cell is counted by the series of these sliced photographs. The stage distribution of a yeast population is estimated from 500 cells at any time. The cell slide specimens are prepared as necessary with bud scars dyed by calcofluor white (1mg/ml) for three minutes. The dead cells are identified by dyeing them by propidium iodide (0.25 μg/ml; 1 minute).

A slide is made by the following procedure. (1) The culture liquid (1 ml) is collected and centrifuged. (2) The calcofluor white liquid (1 mg/ml) is added to the deposit and dyed it for three minutes. (3) The deposit is centrifuged again. (4) The deposit is then washed by distilled water twice. (5) The distilled water of 500 μl is added to the deposit and then the sample is treated by the supersonic wave (65J in 20 seconds). (6) The sample is then centrifuged. (7) From this sample, we prepare the soil suspension of a constant density (100 cells per a single view of fractography). (8) The 17 μl of the soil suspension is dropped on a slide glass. A cover glass is placed on it. Then we apply transparent nail polish on all the edges of cover glass to prevent the sample from drying. (9) For observation of specimens, we use the fluorescent microscope (manufactured by ZEISS) with UV excitation at 630 magnification of oil immersion. (10) For each slide sample, a series of photo images are taken by varying the focus depth of the microscopy at every 0.25 μm. (11) Using these images, we calculate the stage (scar) distributions of cells. We also classify and count the cell phases (G1, S, and G2-M). (12) For some photo images of broken and/or dead cells, a few samples are dyed by propidium iodide (0.25μg/ml) for a minute (double-dye).
