# Supplementary material for: Apoptosis at Inflection Point in Liquid Culture of Budding Yeasts
Source: PLoS One. 2011 Apr 27;6(4):e19224. doi: 10.1371/journal.pone.0019224 (PMC3083425; doi:10.1371/journal.pone.0019224)
Supplement: Text S2 — Simulation procedure. (DOC) [file pone.0019224.s003.doc]

Text S2.

Simulation procedure

We carry out computer simulation in the following procedure: we choose a pair of lattice sites randomly and independently. If the pair is X and O, then the latter empty site becomes X with a growth rate *r*. This is called contact process in chemical reaction. This population growth process can be represented by the chemical reaction formula: X + O → 2X. Let the density of X be *x*. Then the density of empty sites becomes 1- *x*, since the total density is 1 (unity). A density is equal to the population size (the total number of cells) divided by the carrying capacity *K*. If the carrying capacity *K* is fairly large, then the population dynamics is approximated by the differential equation: *dx/dt = rx(1-x).* This is a simple logistic equation.

The current simulation procedure is described as follows:

1. Initially, we distribute three kinds of cells on a one dimensional lattice space (*K* = 4.0x10^4^), where almost all sites are empty. The proportion of daughter and mother is set to 80 to 20 and the total density of cells seeded is 10^2^ (0.25% of the total sites). This proportion is estimated from the growth curves of experimental data.

(2) Reactions (1a)-(1c) are performed in the following two steps.

(i) For the growth process (1a), we randomly choose a lattice site. When the site is

D_i_, then it becomes D_m_ by the growth rate *g*.

(ii) The reproduction processes (1b) and (1c) are performed. We randomly and

independently choose two lattice sites. When the first chosen site is D_m_ or

M_n_, and when the second chosen site is O, then we perform the birth process

(1b) or (1c).

(3) Death process (2) is performed. We randomly choose a lattice site. When the site is

M_n_, then it becomes O by the death rate *m_n_*.

(4) We repeat steps (2) and (3) until the final equilibrium.
